# Intestinal parasitoses in a tertiary-care hospital located in a non-endemic setting during 2006–2010

**DOI:** 10.1186/1471-2334-14-264

**Published:** 2014-05-16

**Authors:** Adriana Calderaro, Sara Montecchini, Sabina Rossi, Chiara Gorrini, Flora De Conto, Maria Cristina Medici, Carlo Chezzi, Maria Cristina Arcangeletti

**Affiliations:** 1 Unit of Microbiology and Virology, Department of Clinical and Experimental Medicine, University of Parma, Viale A. Gramsci 14, 43126 Parma, Italy

**Keywords:** Intestinal parasitosis, Protozoa, Helminths, Epidemiology, Diagnosis, Italy

## Abstract

**Background:**

The aim of this study was to assess the epidemiology of intestinal parasitoses during a 5-year period in patients attending a tertiary-care hospital in a non-endemic setting.

**Methods:**

In the period 2006–2010, 15,752 samples from 8,886 patients with clinically suspected parasitosis were subjected to macroscopic and microscopic examination, to parasitic antigen detection assays, and to cultures for protozoa and nematodes. Real-time PCR assays for the differentiation of *Entamoeba histolytica* and *E. dispar* and for the detection of *Dientamoeba fragilis* were also used.

A statistical analysis evaluating the demographic data of the patients with intestinal parasitic infections was performed.

**Results:**

Intestinal parasitic infections were diagnosed in 1,477 patients (16.6% prevalence), mainly adults and immigrants from endemic areas for faecal-oral infections; protozoa were detected in 93.4% and helminths in 6.6% of the cases, the latter especially in immigrants. *Blastocystis hominis* was the most common intestinal protozoan, and *G. intestinalis* was the most frequently detected among pathogenic protozoa, prevalent in immigrants, males, and pediatric patients. Both single (77.9%) and mixed (22.1%) parasitic infections were observed, the latter prevalent in immigrants.

**Conclusions:**

Despite the importance of the knowledge about the epidemiology of intestinal parasitoses in order to adopt appropriate control measures and adequate patient care all over the world, data regarding industrialized countries are rarely reported in the literature. The data presented in this study indicate that intestinal parasitic infections are frequently diagnosed in our laboratory and could make a contribution to stimulate the attention by physicians working in non-endemic areas on the importance of suspecting intestinal parasitoses.

## Background

Gastrointestinal diseases caused by pathogenic protozoa and helminths are related to a significant amount of morbidity and mortality worldwide, particularly in children; 58 million infections by protozoa were registered every year in children, especially in developing countries as a consequence of the deficiencies in sanitation and the limited access to drinking water [1-3]. Among the population at greatest risk for severe enteric parasitic infections in industrialized countries there are immunocompromised subjects [1,2,4].

In 2004, *Giardia* and *Cryptosporidium* were included in the “Neglected Diseases Initiative” of World Health Organization (WHO) comprising a heterogeneous group of parasitic, bacterial, and viral diseases mostly occurring in developing countries [1].

Although the prevalence of parasitic infections is higher in developing countries, intestinal parasitoses represent frequent diseases also in industrialized ones probably in association with globalization of the food supply, to immigration/adoption from endemic regions, and to travels through the same areas [5]. The risk of contracting parasitic infections, in particular from food, is certainly lower in the developed world than in developing countries due to the accompanying features of poverty. Nevertheless, the relatively mild or non-specific symptoms, the long incubation periods, and the unavailability or the inadequacy of the laboratory methods contribute to underestimate the prevalence of these infections also in industrialized regions [6]. Furthermore, European control strategies are limited and only concern few pathogens, and most of the parasitic diseases are subjected to notification only in some countries [7]. Moreover, the training of physicians is frequently poor about these diseases as they often are neglected [5].

The increased movements from/through run-down areas due to immigration, tourism, work or religious mission could influence the epidemiology of intestinal parasitoses in the area in which our laboratory is located (Italy). Interestingly, the incidence of immigrants in Italy in 2008 was 7.2% [8].

The aim of this study was to assess the epidemiologic picture of parasitic intestinal infections in our area during a 5-year period (2006–2010), by using the data obtained in the routinely diagnostic practice, also in order to define the scenario of such infections in a non-endemic setting for intestinal parasitic diseases.

## Methods

### Study area and population

The study was performed at the University Hospital of Parma, a 1,218-bed tertiary care centre with more than 50,000 admissions registered in the year 2012 [9]. The province of Parma, located in the Northern Italy, has 445,283 inhabitants [10]; the population attending this hospital was estimated in 207,594 inhabitants, 10% of whom were immigrants from developing countries [11].

### Patients, samples and conventional parasitologic assays

The laboratory diagnosis of intestinal parasitosis was performed on 15,722 faecal samples belonging to 8,886 patients, including both hospitalised and outpatients, sent during the period 2006–2010 to our laboratory after a clinical suspicion of intestinal parasitosis. Neither healthy subjects nor people for screening of migrants were included in the study.

7,087 out of 8,886 were Italians and 1,799 immigrants from developing countries, 6,512 were adults, 1,819 children and for 555 the age was unknown, 3,969 were male and 4,917 were female. For the most of the Italian patients a travel history and/or risk factors for infections transmitted by faecal-oral route were not reported or not available. In Table 1 one further partition of Italian and immigrant patients related to age and sex is presented.

**Table 1:** Origin, age, and sex of the patients included in this study

		**Total patients**	**Patients with intestinal parasitoses**^**a**^	**% of patients with intestinal parasitoses on the respective group**	**OR (95% CI)**	***p***
		**8,886**	**1,477**			
Origin	Italians	7,087	892 (63.39%)	12.59% (892/7,087)	3.35 (2.97-3.78)	<0.0025
	Immigrants	1,799	585 (39.61%)	32.52% (585/1,799)		
Age	Adults	6,512	1,210 (81.92%)	18.58% (1,210/6,512)	1.4 (1.21-1.62)	<0.0025
	Children	1,819	255 (17.26%)	14.02% (255/1,819)		
	Age unknown	555	12			
Sex	Males	3,969	692 (46.85%)	17.44% (692/3,969)	1.11 (0.99-1.24)	0.0642
	Females	4,917	785 (53.15%)	15.97% (785/4,917)		

^a^: in brackets the percent proportions of each group of patients calculated on the total of the patients with parasitoses are indicated.

The samples analysed in this study had been submitted to the University Hospital of Parma for routine diagnosis and no approval by the local review committee was required.

The diagnosis of intestinal parasitosis was performed according to standard procedures [12,13] by macroscopic examination of faecal samples and microscopic examination of wet mounts prepared from both fresh and concentrated faeces after formalin-ethyl acetate sedimentation, as previously described [14-16]. Moreover, an immunocromatographic assay (IC) was performed as previously described [15] in order to detect specific antigens of *Cryptosporidium parvum* and *G. intestinalis*. Positive results by IC were confirmed by an immunofluorescence assay performed as previously described [15].

1,652 samples belonging to 906 patients reporting diarrhea, abdominal pain, bloody faeces, eosinophilia, and/or risk factors for parasitic infections, and/or in whose faeces diagnostic stages of intestinal parasites were detected [16], were subjected also to culture for enteric protozoa in Robinson’s medium and to culture for larval stage-nematodes according to standard procedures, as previously described [13,14].

Furthermore, a Scotch test [17] was performed in 116 cases of suspected ossiuriasis in order to detect *Enterobius vermicularis* ova and/or adult worms.

### Molecular assays

The 1,652 faecal samples (906 patients) subjected to cultures were also used to perform PCR assays for the differentiation of *Entamoeba histolytica* and *E. dispar*.

The DNA was extracted partly by using the manual extraction system High Pure PCR Template Preparation Kit (Roche Diagnostics, Mannheim, Germany) as previously described [14], and partly by the automated evolution of the manual system (MagNA Pure LC DNA extraction kit III on the MagNA Pure LC instrument-Roche Diagnostics, Mannheim, Germany) according to the manufacturer’s instructions [16]. The extracted DNA was immediately used for PCR assays or stored at −20°C until analysed.

A conventional PCR assay and its evolution to a FRET (Fluorescence Resonance Energy Transfer) real-time PCR assay detecting and differentiating *E. histolytica* and *E. dispar* were alternatively performed as previously described [14].

Moreover, some of the 1,652 faecal samples (959 specimens belonging to 491 patients, from 2006 to April 2009) were also subjected to a TaqMan real-time PCR assay for the detection of *D. fragilis*, as previously described [18].

**Figure 1:**
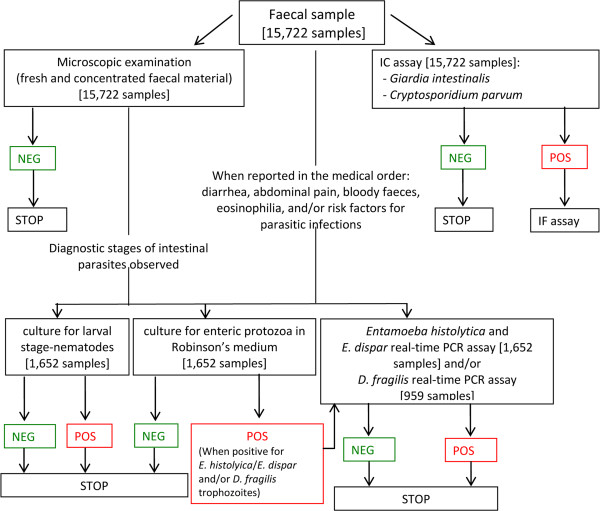
**Flowchart of the algorithm for the diagnosis of intestinal parasitoses used in our laboratory.** Legend: IC = Immunocromatographic assay; IF = Immunofluorescence; POS = positive; NEG = negative.

A flowchart describing the algorithm for the diagnosis of intestinal parasitoses used in our laboratory is reported in Figure 1.

### Statistical analysis

Demographic data (origin, age, and sex) were collected for all the patients and then related to the detected parasitic infections. The statistical significance of the figures of the patients with intestinal parasitic infections into each demographic group was calculated by chi-square test: a p value <0.05, calculated by two-tailed test, was considered significant. Odds ratios (ORs) and 95% confidence intervals (CIs) were calculated in order to evaluate the strength of the associations that emerged. Concerning age data, the group of the patients with age unknown (555 subjects) was not included in the statistical analysis.

## Results

Among the 15,722 samples belonging to 8,886 patients included in this study, intestinal parasites were detected in 2,630 samples belonging to 1,477 patients, corresponding to a 16.6% prevalence of patients with intestinal parasitoses. A statistical analysis concerning the patients with intestinal parasitoses diagnosed in this study in association with origin, age, and sex is reported in Table 1.

On the total of the parasites detected in this study (1,915) in the samples belonging to the 1,477 patients with parasitic infections either single or in mixed combinations, 1,789 (93.4%) were protozoa and 126 (6.6%) were helminths (Table 2).

**Table 2:** Parasites found in faeces of the 1,477 infected patients with mentions to demographic data

**Parasite**	**No. of parasites**^**a**^	**Age of patients**					**Origin of patients**				**Sex**			
		**Adults**^**b**^	**Children**^**b**^	**Unknown**	**OR (95% CI)**	***p***	**Italians**^**b**^	**Immigrants**^**b**^	**OR (95% CI)**	***p***	**Male**^**b**^	**Female**^**b**^	**OR (95% CI)**	***p***
**Protozoa**	**1,789**	**1,417 (21.76%)**	**357 (19.63%)**	**15**	**1.14 (1.0-1.30)**	**0.0494**	**1,014 (14.31%)**	**775 (43.08%)**	**4.53 (4.04-5.08)**	**0**	**864 (21.67%)**	**925 (18.81%)**	**1.20 (1.08-1.33)**	**0.0005**
*Blastocystis hominis*	1,234 (13.89%)	1,046 (16.06%)	178 (9.79%)	10	1.76 (1.49-12.09)	<0.0025	760 (10.72%)	474 (26.35%)	2.98 (2.62-3.39)	<0.0025	558 (14.06%)	676 (13.75%)	1.03 (0.91-1.16)	0.6737
*Giardia intestinalis*	168 (1.89%)	101 (1.55%)	65 (3.75%)	2	2.35 (1.71-3.23)	<0.0025	85 (1.2%)	83 (4.61%)	3.98 (2.93-5.42)	<0.0025	101 (2.54%)	67 (1.36%)	1.89 (1.38-2.58)	<0.0025
*Dientamoeba fragilis*	149 (1.68%)	97 (1.49%)	50 (2.75%)	2	1.87 (1.32-2.64)	0.0003	72 (1.02%)	77 (4.28%)	4.36 (3.15-6.03)	0	77 (1.94%)	72 (1.46)	1.33 (0.96-1.84)	0.0825
*Entamoeba coli*	148 (1.67%)	102 (0.71%)	46 (5.61%)	0	8.35 (5.87-11.88)	0	45 (0.64%)	103 (5.73%)	9.50 (6.67-13.54)	0	69 (1.74%)	79 (1.61%)	1.08 (0.78-1.50)	0.6293
*Entamoeba dispar*	69 (0.78%)	58 (0.15%)	10 (3.19%)	1	17.85 (9.56-33.30)	0	36 (0.51%)	33 (1.83%)	3.66 (2.28-5.89)	0	44 (1.11%)	25 (0.51%)	2.19 (1.34-3.59)	0.0014
*Cryptosporidium* spp*.*	17 (0.19%)	11 (0.09%)	6 (0.6%)	0	6.60 (2.44-17.86)	0	14 (0.20%)	3 (0.17%)	1.18 (0.34-4.13)	0.7896	11 (0.28%)	6 (0.12%)	2.27 (0.84-6.16)	0.0962
*Entamoeba histolytica*	4 (0.05%)	2 (0.03%)	2 (0.11%)	0	3.58 (0.50-25.45)	0.1726	2 (0.03%)	2 (0.11%)	0.25 (0.04-1.80)	0.1385	4 (0.10%)	0	/	0.026
**Helminths**	**126**	**86 (1.32%)**	**39 (2.14%)**	**1**	**1.64 (1.12-2.40)**	**0.0107**	**39 (0.55%)**	**87 (4.84%)**	**9.18 (6.27-13.45)**	**0**	**70 (1.76%)**	**56 (1.13%)**	**1.56 (1.09-2.22)**	**0.0133**
*Strongyloides stercoralis*	36 (0.40%)	32 (0.49%)	4 (0.22%)	0	2.24 (0.79-6.34)	0.1186	7 (0.10%)	29 (1.61%)	0.36 (0.15-0.87)	0.0157	24 (0.6%)	12 (0.24%)	7.46 (1.24-4.98)	0.0078
*Enterobius vermicularis*	35 (0.39%)	18 (0.28%)	17 (0.93%)	0	3.40 (1.75-6.62)	0.0001	19 (0.27%)	16 (0.89%)	3.34 (1.71-6.50)	0.0002	9 (0.23%)	26 (0.53%)	2.34 (1.09-5.00)	0.0238
*Hymenolepis nana*	12 (0.14%)	4 (0.06%)	8 (0.44%)	0	7.19 (2.16-29.90)	0.0002	0	12 (0.67%)	/	0	6 (0.15%)	6 (0.12%)	1.24 (0.40-3.85)	0.7099
*Taenia saginata*	12 (0.14%)	11 (0.17%)	1 (0.05%)	0	3.08 (0.40-23.84)	0.2573	8 (0.11%)	4 (0.22%)	1.97 (0.59-6.56)	0.2589	8 (0.2%)	4 (0.08%)	2.48 (0.75-8.24)	0.1250
*Taenia* spp*.*	9 (0.1%)	8 (0.12%)	1 (0.05%)	0	2.24 (0.28-17.89)	0.4359	2 (0.03%)	7 (0.39%)	13.84 (2.87-66.67)	0	5 (0.13%)	4 (0.08%)	1.55 (0.42-5.77)	0.5109
*Ascaris lumbricoides*	9 (0.1%)	5 (0.08%)	4 (0.22%)	0	2.87 (0.77-10.69)	0.1004	3 (0.04%)	6 (0.33%)	7.90 (1.24-79.42)	0.0005	8 (0.2%)	1 (0.02%)	9-93 (1.97-31.63)	0.0076
*Trichuris trichiura*	6 (0.07%)	5 (0.08%)	1 (0.05%)	0	1.40 (0.16-11.9)	0.7592	0	6 (0.33%)	/	0	5 (0.13%)	1 (0.02%)	6.20 (0.72-53.10)	0.0567
*Ancylostoma duodenale*	4 (0.05%)	2 (0.03%)	1 (0.05%)	1	1.79 (0.16-19.76)	0.6297	0	4 (0.22%)	/	0.0001	3 (0.08%)	1 (0.02%)	3.72 (0.39-35.79)	0.2222
*Dicrocoelium dendriticum*	2 (0.02%)	1 (0.02%)	1 (0.05%)	0	3.58 (0.22-57.2)	0.3349	0	2 (0.11%)	/	0.0050	1 (0.26%)	1 (0.02%)	1.24 (0.08-19.81)	0.8794
*Schistosoma mansoni*	1 (0.01%)	0	1 (0.05%)	0	/	0.0585	0	1 (0.05%)	/	0.0472	1 (0.26%)	0	/	0.2657
**Total**	**1,915**	**1,503**	**396**	**16**			**1,053**	**862**			**934**	**981**		

^a^: in brackets the percentages on 8,886 total patients are reported, calculated as the occurrence of the infections by protozoa and those by helminths in the population studied, not taking into account their involvement either in single or in mixed infections but considering the parasitoses (and subsequently the respective parasites involved) once at a time.

^b^: in brackets the percent proportions of each group of patients calculated on the respective total are indicated.

In Table 2 the percentages expressing the occurrence of the infections by protozoa and those by helminths in the population studied were reported, not taking into account their involvement either in single or in mixed infections but considering the parasitoses (and subsequently the respective parasites involved) once at a time. In Table 3 the prevalence of infections detected by the different diagnostic methods was reported.

**Table 3:** Parasites detected by different diagnostic methods in the samples of the 1,477 infected patients

**Parasite**	**No. of parasites**	**Diagnostic methods**								
		**M**^**a **^**only**	**C**^**b **^**only**	**Both M and C**^**b**^	**PCR**^**c**^	**Both M and PCR**^**c**^	**Both C and PCR**^**c**^	**M, C, and PCR**^**c**^	**Both IC and M**^**a**^	**Both IC and IF**^**a**^
**Protozoa**	**1,789**									
*Blastocystis hominis*	1,234	502 (5.64%)	55 (6.07%)	677 (74.2%)	-	-	-	-	-	-
*Giardia intestinalis*	168	0	-	-	-	-	-	-	163 (1.83%)	5 (0.06%)
*Dientamoeba fragilis*	149	41 (0.46%)	3 (0.33%)	0	60 (12.22%)	3 (0.61%)	39 (7.94%)	3 (0.61%)	-	-
*Entamoeba coli*	148	110 (1.24%)	0	38 (4.19%)	-	-	-	-	-	-
*Entamoeba dispar*	69	-	-	-	7 (0.77%)	26 (2.87%)	5 (0.55%)	31 (3.42%)	-	-
*Cryptosporidium* spp*.*	17	-	-	-	-	-	-	-	-	17 (0.19%)
*Entamoeba histolytica*	4	-	-	-	3 (0.33%)	1 (0.11%)	0	0	-	-
		**M**^**a,e **^**only**	**C**^**b,f **^**only**	**Both M and C**^**b,f**^	**ST**^**d **^**only**	**Both M and ST**^**d,e**^	**MA**^**a **^**only**	**Both M and MA**^**a,g**^		
**Helminths**	**126**									
*Strongyloides stercoralis*	36	-	17 (1.88%)	29 (0.33%)	-	-	-	-		
*Enterobius vermicularis*	35	27 (0.3%)	-	-	6 (5.17%)	2 (1.72%)	-	-		
*Hymenolepis nana*	12	12 (0.13%)	-	-	-	-	-	-		
*Taenia saginata*	12	0	-	-	-	-	3 (0.13%)	9 (0.10%)		
*Taenia* spp*.*	9	7 (0.08%)	-	-	-	-	0	2 (0.02%)		
*Ascaris lumbricoides*	9	7 (0.08%)	-	-	-	-	1 (0.01%)	1 (0.01%)		
*Trichuris trichiura*	6	6 (0.07%)	-	-	-	-	0	0		
*Ancylostoma duodenale*	4	0	4 (0.44%)	-	-	-	-	-		
*Dicrocoelium dendriticum*	2	2 (0.02%)	-	-	-	-	-	-		
*Schistosoma mansoni*	1	1 (0.01%)	-	-	-	-	-	-		
**Total**	**1,915**									

M: Microscopic examination; C: Culture; IC: Immunocromatographic assay for the detection of specific antigens of *Cryptosporidium parvum* and *Giardia intestinalis*; IF: Immunofluorescence assay to detect *Cryptosporidium* spp*.* oocysts and *G. intestinalis* cysts; ST: Scotch Test; MA: Macroscopic examination; − : Method not applicable.

^a^: in brackets the % on 8,886 patients are reported.

^b^: in brackets the % on 906 patients are reported.

^c^: in brackets the % on 906 and on 491 patients are reported, when PCR assays for the differentiation of *E. histolytica* and *E. dispar* and the real-time PCR assay for the identification of *D. fragilis* were respectively applied.

^d^: in brackets the % on 116 patients are reported.

^e^: microscopic examination is referred to ova identification.

^f^: microscopic examination and culture are referred to larvae identification.

^g^: microscopic examination is referred to ova identification and macroscopic identification of adult stages.

The most commonly detected intestinal protozoan was *Blastocystis hominis*, a parasite whose pathogenic role is still controversial, and, regarding the pathogenic ones, *G. intestinalis* was the most frequently detected among protozoa, and *S. stercoralis* among helminths.

The occurrence of the parasites (reported as %) found in the patients with intestinal parasitic infections and a statistical analysis with mention to origin, age, and sex are reported in Table 2.

Out of the total of the 1,477 patients with intestinal parasitoses, single parasitic infections were observed in 1,150 cases corresponding to 77.9% (Table 4), 65.91% Italian patients and 34.09% immigrant patients. The frequency of single infections in association with origin was 85% (758/892) in Italians and 97% (392/585) (OR 2.79; CI 2.16-3.58; *p* = 0) in immigrants.

**Table 4:** Patients with intestinal parasitic infections caused by a single parasite with mentions to demographic data

	**Total**	**Age**			**Origin**		**Sex**	
		**Adults**	**Children**	**Unknown**	**Italians**	**Immigrants**	**Male**	**Female**
*Blastocystis hominis*	943	834	103	6	638	305	407	536
*Giardia intestinalis*	78	48	30	0	43	35	51	27
*Dientamoeba fragilis*	29	22	7	0	20	9	12	17
*Entamoeba coli*	26	17	9	0	12	14	10	16
*Enterobius vermicularis*	22	12	10	0	14	8	7	15
*Cryptosporidium* spp*.*	15	11	4	0	13	2	10	5
*Entamoeba dispar*	13	11	1	1	7	6	7	6
*Strongyloides stercoralis*	10	10	0	0	2	8	7	3
*Taenia saginata*	7	7	0	0	6	1	4	3
*Taenia* spp.	2	2	0	0	0	2	1	1
*Ascaris lumbricoides*	2	1	1	0	2	0	2	0
*Entamoeba histolytica*	1	1	0	0	1	0	1	0
*Ancylostoma duodenale*	1	0	0	1	0	1	1	0
*Hymenolepis nana*	1	0	1	0	0	1	0	1
**Total**	**1,150**	**976**	**166**	**8**	**758**	**392**	**520**	**630**

Mixed parasitic infections were observed in 327 cases corresponding to 22.1% out of the total of the patients with intestinal parasitic infections, 41% Italian patients and 59% immigrant patients. The frequency of mixed infections in association with origin was 15.02% (134/892) in Italians and 32.9% (193/585) in immigrants (OR 2.79; CI 2.16-3.58; *p* < 0.0025).

Protozoa were present in single infections in 74.8% of the cases and in mixed infections in 25.2% of the cases.

Helminths were present in single infections in 35.71% of the cases and in mixed infections in 64.29% of the cases.

Among mixed infections the most frequent combinations were *B. hominis* and *Entamoeba coli* (56), *B. hominis* and *D. fragilis* (54), *B. hominis* and *G. intestinalis* (44), *B. hominis* and *E. dispar* (20), *B. hominis*, *E. coli*, and *D. fragilis* (18).

The majority of patients with mixed infections (235) had a parasitosis caused by 2 parasites. Seventy-seven patients had a mixed infection caused by 3 parasites.

Eleven patients presented with 4 simultaneous parasitic infections, 3 patients with 5 simultaneous parasitic infections, and 1 patient with 6 simultaneous parasitic infections, both by protozoa and helminths.

## Discussion

Many epidemiological data on the diffusion and the prevalence of intestinal parasitoses in humans are available for developing areas, but in industrialized countries intestinal parasitoses are usually not notified and few data are reported in the literature. In Italy recent epidemiological reports are restricted to the analysis of few parasitic agents i.e. [19] or to a selected population i.e. [8,20], except for a study describing the presence of intestinal parasites isolated in a large teaching hospital located in Rome during a period of 32 months [5].

This is the first study reporting the occurrence of intestinal parasitic infections in a non-endemic setting investigated by using the data obtained by parasitological examination daily performed on samples belonging to patients with the clinical suspicion of parasitosis based on several abdominal disorders, admitted to our University Hospital during a 5-year period. These data allowed obtaining the actual scenario of our area in the light of the continuous changes in the composition of the population and in the habits in order to make a contribution to stimulate the attention by both physicians and microbiologists on the importance of suspecting and diagnosing intestinal parasitoses.

Furthermore the data could be representative of both the whole Italian and European scenarios, that are likely comparable to our setting in terms of risk of transmission of intestinal parasites by faecal-oral route.

The prevalence of intestinal parasitic infections detected in this study (16.6%) was unexpectedly high in a non-endemic area for infections with a parasitic aetiology transmitted by faecal-oral route. As a matter of fact, our laboratory receives samples from individuals who immigrate from or travel through developing countries and presenting risk factors for acquiring parasitic infections including malaria [21], proving that human flows to our area are related to importation of parasitic agents. Interestingly, 6 patients with intestinal parasitic infections (4 cases by *B. hominis*, 1 case by *E. dispar* and *G. intestinalis*, 1 case by *A. lumbricoides* and *T. trichiura*) reported also concomitant infections by plasmodia [22]. Among the group of the patients included in this study a significant difference in the prevalence of parasitic infections emerged in adults (1.4 times higher than in children), and in immigrants (3.35 times higher than in Italians). The difference between males and females was not remarkable.

In our study *B. hominis*, often reported as the most commonly detected organism in parasitological surveys [5], was the most frequently detected among intestinal protozoa. In our setting the prevalence of *B. hominis* was significantly higher in immigrants than in Italians (about 3 times) and in adults than in children (1.76 times) all having intestinal symptoms. Despite its role in pathogenesis is controversial [23], epidemiological data about the prevalence of *B. hominis* in the analysed population were essential to state that a faecal-oral route subsists in our area and this study made a contribution to unravel this scenario.

*G. intestinalis* is known as the most common enteric protozoan pathogen of humans, domestic and wildlife animals, having a more relevant prevalence in warm climate and in children [15], particularly those living in developing countries and in disadvantaged community settings [24]. In Italy giardiasis is a not notifiable disease and prevalence data are based on specialised studies reporting percentages of infection ranging from 0.9% to 2.41% [19]. In this study, this epidemiological trend was confirmed being *G. intestinalis* the second parasite detected in the analysed population with a prevalence of 1.89%, similarly to previously reported data [i.e. 5]. The prevalence of *G. intestinalis* was significantly higher in immigrants than in Italians (about 4 times) and in males than in females (1.89 times). Interestingly, as expected, giardiasis was more frequent in paediatric patients than in adults (2.35 times).

In general, the results reported in this study showed that regarding the parasitoses by protozoa the infection rate was significantly higher in males than in females (1.20 times) and in immigrants than in Italians (4.53 times). Concerning the frequency of the protozoa, the infection rate was significantly higher in children than in adults, except for *B. hominis*.

It is noteworthy that in this study a whole of 73 patients was diagnosed with an infection by the pathogenic species *E. histolytica* or by the non-pathogenic species *E. dispar*, microscopically not distinguishable: in these cases the differentiation at the species level was accomplished by PCR that revealed 4 infections by *E. histolytica* and 69 by *E. dispar* (corresponding to a prevalence of 0.44% and 7.61%, respectively, calculated on the total of the patients with a targeted suspicion of amoebiasis and whose samples were submitted to PCR assay). This demonstrated that PCR was in our hands an essential tool which allowed focusing on *E. histolytica* infections with the administration of a targeted therapy only in those cases and avoiding the treatment in the patients with *E. dispar* infections.

In our study helminthic infections resulted lower in frequency than protozoan ones (93.4% vs. 6.6% on the total of detected parasites); the higher prevalence of parasitoses and in particular of helminthiasis in immigrants (2.23 times as compared to that of Italians) is not unexpected since it is known that helminthic infections are more frequent in the population living in developing countries and in immigrants from those areas [25]. Unlike the most of epidemiologic research focused on the occurrence of helmintic infections depending on age, revealing that changes with age in the average intensity of infection tend to be convex, rising in childhood and declining in adulthood [5], interestingly our data did not show any difference in the prevalence of helminthiasis concerning the age group of patients.

In our setting mixed parasitic infections proved to weigh considerably on the global epidemiology (22.7%), especially in the population from developing countries. These data confirmed those reported in other industrialized countries such as North America and Europe where parasitic infections are most prevalent within immigrant and refugee communities [26]. Furthermore, when evaluating the association of the origin of the patients and the occurrence of mixed infections, the higher frequency (2.79 times) in immigrants compared to that of Italians resulted statistically significant.

## Conclusions

Knowledge about the epidemiology of parasitic infections becomes an essential tool in non-endemic areas in order to adopt appropriate control measures and adequate patient care, underlining that intestinal parasitoses should be considered in the differential diagnosis of gastrointestinal diseases. In this light, in our experience the use of a combination of different diagnostic methods was demonstrated to be necessary in order to ensure a prompt, accurate, and complete diagnosis of intestinal parasitoses including mixed infections: the conventional standard procedures including cultures for protozoa and helminthic larvae enable the detection of the different parasites of medical interest as well as molecular assays allow to focus on parasites otherwise not distinguishable (*E. histolytica* and *E. dispar*) or difficult to reveal (*D. fragilis*).

The data reported herein could be useful for physicians working in non-endemic areas with the aim of increasing their attention during the anamnesis about the concrete possibility of intestinal parasitoses in patients reporting signs and symptoms, and/or risk factors consistent with this suspicion.

Moreover, the data reported in this study could be also useful for parasitologists in order to obtain information suitable to plan the adoption of appropriate tools to achieve an accurate laboratory diagnosis of parasitic infections.

## Abbreviations

IC: Immunocromatographic assay; FRET: Fluorescence resonance energy transfer; OR: Odds ratio; CI: Confidence interval.

## Competing interests

The authors declare that they have no competing interests.

## Authors’ contributions

AC, SM, SR, CG conceived the study. AC, SM, SR, CG designed the study protocol. AC, CC carried out the clinical assessment. AC, CC, CG, SR, FD, MCM, MCA carried out the analysis and interpretation of the data. AC, CG, SM drafted the manuscript. AC, CC critically revised the manuscript for intellectual content. All authors read and approved the final manuscript: AC, CC, MCM, FD, MCA are guarantors of the paper.

## Pre-publication history

The pre-publication history for this paper can be accessed here:

http://www.biomedcentral.com/1471-2334/14/264/prepub
